# Molecular basis for alternative charge separation pathways in type-II photosynthetic reaction centers

**DOI:** 10.1093/pnasnexus/pgag111

**Published:** 2026-04-09

**Authors:** Tomoyasu Noji, Keisuke Saito, Hiroyuki Tamura, Hiroshi Ishikita

**Affiliations:** Department of Applied Chemistry, The University of Tokyo, 7-3-1 Hongo, Bunkyo-ku, Tokyo 113-8654, Japan; Research Center for Advanced Science and Technology, The University of Tokyo, 4-6-1 Komaba, Meguro-ku, Tokyo 153-8904, Japan; Department of Applied Chemistry, The University of Tokyo, 7-3-1 Hongo, Bunkyo-ku, Tokyo 113-8654, Japan; Research Center for Advanced Science and Technology, The University of Tokyo, 4-6-1 Komaba, Meguro-ku, Tokyo 153-8904, Japan; Department of Applied Chemistry, The University of Tokyo, 7-3-1 Hongo, Bunkyo-ku, Tokyo 113-8654, Japan; Research Center for Advanced Science and Technology, The University of Tokyo, 4-6-1 Komaba, Meguro-ku, Tokyo 153-8904, Japan; Department of Applied Chemistry, The University of Tokyo, 7-3-1 Hongo, Bunkyo-ku, Tokyo 113-8654, Japan; Research Center for Advanced Science and Technology, The University of Tokyo, 4-6-1 Komaba, Meguro-ku, Tokyo 153-8904, Japan

**Keywords:** alternative charge separation pathway, bacteriochlorophyll, methyl-keto (acetyl) group, electron transfer, asymmetry, photosynthetic reaction center

## Abstract

In photosynthetic reaction centers from purple bacteria, bacteriochlorophyll *a* (the special pair P_A_P_B_ and accessory bacteriochlorophyll B_A_) and bacteriopheophytin *a* (H_A_) form the active electron-transfer branch and uniquely contain a polar methyl-keto (3-acetyl) group. However, despite its importance in defining local polarity, even the currently available highest-resolution structures (∼2 Å) cannot unambiguously distinguish the methyl carbon from the keto oxygen, limiting insight into its functional role. Here, we investigate how the methyl-keto orientations of the P_B_ and H_A_ cofactors influence the energetics of charge-separated intermediates, using a quantum mechanical/molecular mechanical approach. We identify two kinetically isolated, metastable methyl-keto conformations of P_B_, Tyr-OH…B_A_ and Tyr-OH…P_B_, each associated with a distinct charge-separation pathway: the canonical [P_A_P_B_]* → [P_A_P_B_]^•+^B_A_^•−^ and alternative B_A_* → B_A_^•+^H_A_^•−^ pathways, respectively. For H_A_, methyl-keto reorientation stabilizes H_A_^•−^ when forward transfer to the primary quinone (Q_A_) is inhibited. These results show that distinct methyl-keto conformations selectively tune charge-separation routes while also contributing to the oxidative robustness of bacteriochlorin macrocycles.

Significance statement Photosynthetic charge separation is often viewed as following a single, well-defined pathway, yet alternative routes have long been observed without a clear molecular origin. Here, we show that the orientation of a single polar methyl-keto group in reaction-center cofactors, whose configuration cannot be reliably resolved even in currently available high-resolution structures, can selectively control which charge-separation pathway is favored. This effect arises from orientation-dependent local electrostatic interactions that selectively stabilize different charge-separated states. Our results demonstrate that subtle, unresolved local structural features can govern electron transfer without requiring global protein rearrangements, providing a simple physical basis for functional flexibility in photosynthetic reaction centers.

## Introduction

**Figure 1:**
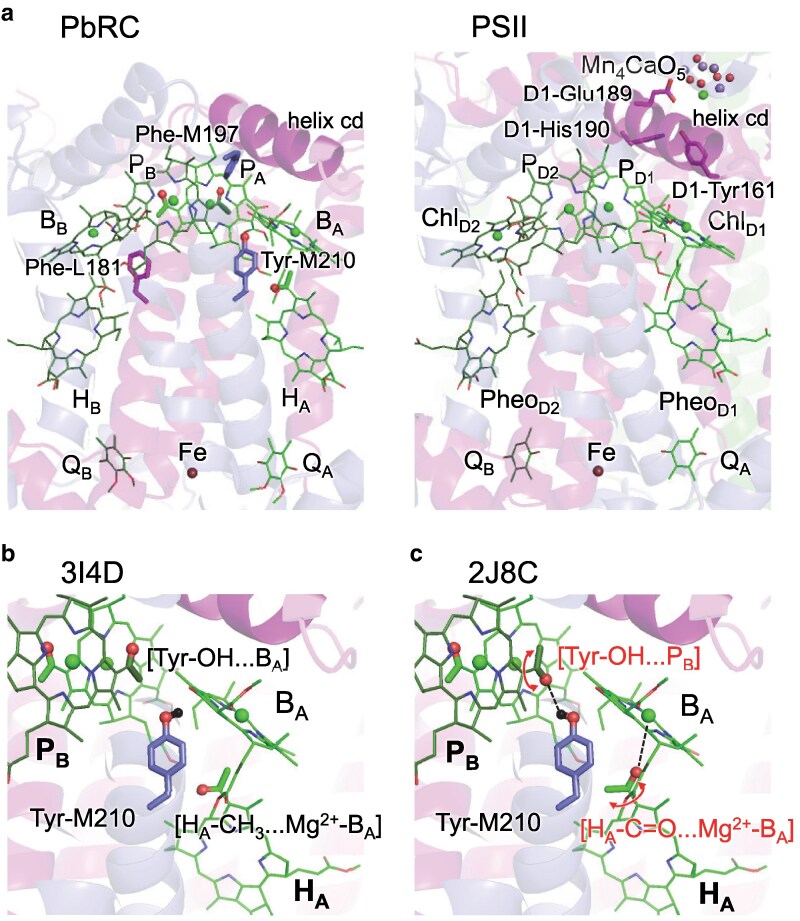
Arrangement of cofactors in type-II reaction centers. a) Overall cofactor organization in PbRC (PDB code: 3I4D) and PSII (PDB code: 3WU2), highlighting the helix cd region and the active (light green, eg P_A_) and inactive (dark green, eg P_B_) electron-transfer branches. The Tyr-M210/Phe-L181 pair is shown as stick models. b) PbRC structure at 2.01 Å resolution (PDB code: 3I4D), in which P_B_ adopts the **Tyr-OH…B_A_** conformation and H_A_ adopts the **H_A_–CH_3_…Mg^2+^–B**_A_ conformation. c) PbRC structure at 1.87 Å resolution (PDB code: 2J8C), in which P_B_ adopts the **Tyr-OH…P_B_** conformation and H_A_ adopts the **H_A_–C=O…Mg^2+^–B**_A_ conformation. Red balls indicate polar oxygen atoms of the methyl-keto (acetyl) groups and the hydroxyl oxygen of Tyr-M210. The black ball indicates the hydroxyl hydrogen of Tyr-M210. Key electrostatic interactions are indicated by black dotted lines. Red circled arrows indicate the rotated methyl-keto conformations in the 1.87 Å structure relative to the 2.01 Å structure.

In photosynthetic reaction centers, light absorption and the subsequent charge separation and electron transfer constitute the core processes of energy conversion. These reaction centers are typically composed of two similar or identical protein subunits, forming either heterodimeric or homodimeric complexes. Accordingly, they exhibit either pseudo-*C*_2_ or true *C*_2_ symmetry axes aligned with the transmembrane direction, resulting in two seemingly symmetric electron-transfer branches. Type II reaction centers, such as photosystem II (PSII) and the reaction center of purple bacteria (PbRC), possess heterodimeric structures with pseudo-*C*_2_ symmetry and share notable similarities in the overall architecture of their electron-transfer cofactors (1–4). Each contains a pair of (bacterio)chlorophylls, P_D1_/P_D2_ in PSII and P_A_/P_B_ in PbRC, accessory (bacterio)chlorophylls (Chl_D1_/Chl_D2_ in PSII; B_A_/B_B_ in PbRC), (bacterio) pheophytins (Pheo_D1_/Pheo_D2_ in PSII; H_A_/H_B_ in PbRC), and quinones (Q_A_ and Q_B_) aligned along the two branches (Fig. 1a).

In both PSII and PbRC, electron transfer proceeds predominantly along a single branch: the D1 branch in PSII and the A branch in PbRC, revealing functional asymmetry despite apparent structural symmetry (5–7). However, the mechanisms underlying this asymmetry are fundamentally different. In PbRC, strong electronic coupling [∼110 meV (8)] between P_A_ and P_B_ leads to the formation of the special pair, which serves as the primary electron donor. Primary charge separation in PbRC has been analyzed theoretically for decades, including electrostatic/energetic control by the protein matrix and electron-transfer theory descriptions of the [P_A_P_B_]-B_A_ pathway (9–12). In contrast, in PSII, the coupling between P_D1_ and P_D2_ is considerably weaker [∼10 meV (8)], and the lowest excitation energy site is monomeric Chl_D1_. As a result, the initial charge separation in PSII involves Chl_D1_ rather than the P_D1_/P_D2_ pair.

This divergence ultimately originates from the significant difference in the protein electrostatic environment between PSII and PbRC. In PSII, the requirement to split water and evolve oxygen necessitates the incorporation of numerous charged and polar residues to stabilize the Mn_4_CaO_5_ cluster and to guide protons, produced as a byproduct, along the associated exit pathways (13). For example, the luminal α-helix cd (D1-176–190) near Chl_D1_ provides D1-Glu189, a ligand to the Mn_4_CaO_5_ cluster, and D1-His190, which forms a low-barrier H-bond with D1-Tyr161 (14), making the Tyr-His pair (TyrZ) a redox-active intermediate that accepts electrons from substrate water molecules at the cluster. This cd helix is four residues longer than the corresponding helix in PbRC (L152–162), contributing to an upshift in the redox potential of P_D1_ and thereby enhancing its oxidation power (15). In PbRC, the cd helix provides an axial ligand (His-L153) to B_A_. However, the corresponding histidine ligand to Chl_D1_ is absent in PSII. Furthermore, PbRC lacks the charged residues necessary for water oxidation, resulting in a largely charge-neutral environment near its bacteriochlorophylls. Thus, remarkably, the asymmetry in the activity of electron transfer branches in PbRC is achieved predominantly by charge-neutral residues.

These charge-neutral residues are largely conserved between subunit L and M of the transmembrane region of PbRC. Among such residue pairs, some exceptions, in particular, Tyr-M210, positioned near B_A_ and replaced with nonpolar Phe-L181 near B_B_, has been suggested to play a particularly important role in the initial charge separation involving the B_A_^•−^ state (10, 11, 16–18). As the closest polar residue to B_A_, Tyr-M210 substantially increases the redox potential of B_A_, stabilizing the B_A_^•−^ state and facilitating electron transfer along the A-branch (8, 17). Mutations of Tyr-M210 to phenylalanine significantly slows the initial electron transfer (19) [eg from 3.5 ps to 16 ps (20)].

Nevertheless, the asymmetry in electron-transfer activity between the two branches cannot be fully explained by the polarity difference of this single Tyr-M210/Phe-L181 pair. In PbRC, this issue has been explored through mutagenesis, including the replacement of His-M202 (His-M200 in *Blastochloris viridis* PbRC), the axial ligand to P_B_, with leucine, resulting in a bacteriochlorophyll/bacteriopheophytin heterodimer special pair, or swapping the Tyr-M210/Phe-L181 pair near B_A_/B_B_ to phenylalanine/tyrosine, have been investigated in this context (6, 7, 21, 22). Although such modifications can increase the probability of initial charge separation via B_B_^•−^ along the B-branch, electron transfer along the B-branch remains limited. Even with multiple mutations designed to alter this asymmetry, the B-branch contributes only a few percent (∼6%) to total electron transfer (6, 7). These extensive mutagenesis studies indicate that, beyond local side chains near the bacteriochlorophylls, additional and as yet unidentified factors contribute to the functional asymmetry of the two branches.

Remarkably, bacteriochlorophyll and bacteriopheophytin employed in PbRC contain a methyl-keto group (3-acetyl, –COCH_3_) on its chlorin ring. Given the proximity of the P, B, and H, their methyl-keto groups are polar groups that are closest to and potentially most crucial to the neighboring chlorin cofactors in the electron transfer branches in the transmembrane region. Spectroscopic studies have suggested that the H_A_^•−^ state can exist in two energetically distinct conformations due to reorientation of this methyl-keto group (23). A 180° twist of the methyl-keto group can modulate the redox potential by ∼50 mV (24). Since the chlorophylls employed in PSII lack a corresponding polar group, this unique feature of bacteriochlorophyll may be relevant to the electron-transfer process specific to PbRC. However, unambiguous assignment of the methyl-keto group orientation in crystal structures remains challenging, even in the most refined PbRC datasets resolved at ∼2 Å resolution (17, 24) (Fig. 1b and c). Notably, different PbRC crystal structures report different orientations of the P_B_ methyl-keto group (Table S1, Fig. S1). While such variability may partly reflect limitations in modeling small functional groups that lack a strong, unique H-bond partner, it may also indicate the presence of multiple energetically accessible conformations. Consistent with this view, crystal structures of light-harvesting antenna proteins have revealed deviations in the orientation of the methyl-keto group of bacteriochlorophyll *a*, highlighting the intrinsic conformational flexibility of this substituent (25). In addition, a related issue has been analyzed for the formyl group of chlorophyll *f* in photosystem I acclimated to far-red light, where conformational heterogeneity of the formyl group complicates its structural identification in cryo-electron microscopy maps (26).

For P_B_, crystal structures reveal two distinct orientations of the methyl-keto group. In one conformation [eg PDB code: 3I4D, 2.01 Å resolution (27); Fig. 1b], the methyl carbon of the P_B_ methyl-keto group faces Tyr-M210, orienting the hydroxyl group of Tyr-M210 toward B_A_ (ie **Tyr-OH…B_A_ conformation**). In the other conformation [eg PDB code: 2J8C, 1.87 Å resolution (28); Fig. 1c], the keto oxygen of P_B_ faces Tyr-M210, forming an H-bond between the P_B_ keto oxygen and Tyr-M210 (ie **Tyr-OH…P_B_ conformation**) (Table S1, Fig. S1). In contrast, all reported crystal structures indicate that the methyl-keto group of P_A_ consistently adopts a single, well-defined conformation, in which the keto oxygen forms an H-bond with His-L168. The corresponding residue on the M subunit is Phe-M197: the absence of an H-bond donor at this position allows P_B_ to adopt two distinct methyl-keto conformations, as demonstrated in the F(M197)H mutant (29).

Among other chlorin cofactors, B_A_ and H_A_ also possess methyl-keto groups. Specifically, the methyl-keto group of H_A_ is situated near the Mg^2+^ ion of B_A_, approximately perpendicular to the chlorin ring of B_A_ (Fig. 1). In some structures [eg PDB code: 3I4D, 2.01 Å resolution (27)], the methyl carbon of the H_A_ methyl-keto group is oriented toward Mg^2+^ of B_A_ (ie **H_A_–CH_3_…Mg^2+^–B_A_ conformation**; Fig. 1b), while in other structures [eg PDB code: 2J8C, 1.87 Å resolution (28)], the keto oxygen is oriented toward Mg^2+^, mimicking an axial-ligand-like geometry (ie **H_A_–C=O…Mg^2+^–B_A_ conformation**; Fig. 1c). Since no H-bond partner is available near the H_A_ methyl-keto group, both conformations are possible in PbRC crystal structures (Table S1, Fig. S1).

While methyl-keto group orientations are known to affect the electronic structure and spectroscopic properties of bacteriochlorophyll *a* in the absence of a protein environment (25, 30), comparatively little is known about their impact in the PbRC protein environment (23, 24). In PbRC, not only nearby polar residues but also polar functional groups of neighboring cofactors can interact with a given chlorin, potentially modulating its electronic properties (24). Indeed, the three polar groups, [P_B_ methyl-keto]…[Tyr-M210 hydroxyl]…[H_A_ methyl-keto], are approximately aligned along the same axis bridging [P_A_P_B_] and H_A_, corresponding to the shortest edge-to-edge distance (12) between cofactors (Fig. 1). This spatial arrangement suggests that these groups may collectively define a relevant electron-transfer route during the initial charge-separation process.

Here, to gain insight into how the orientation of such polar groups, specifically those in P_B_ and H_A_ adjacent to B_A_ and B_A_, respectively, impacts the initial charge-separation process, we examined the charge-separation energetics, using a quantum mechanical/molecular mechanical (QM/MM) approach that explicitly incorporates the full protein environment.

## Results

**Figure 2:**
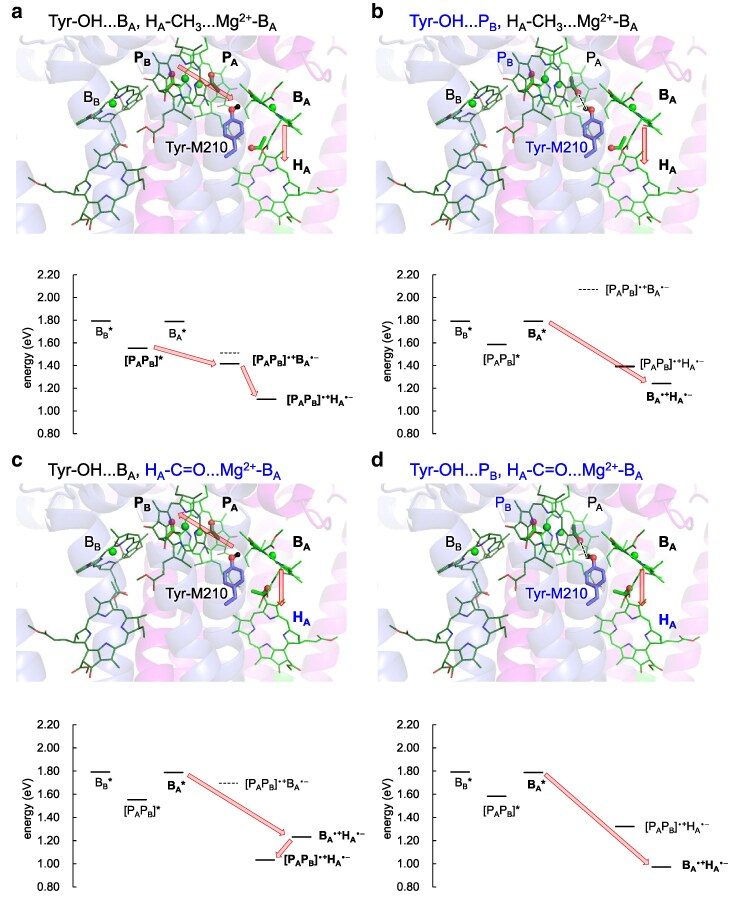
Methyl-keto orientations of P_B_ and H_A_ (top) and associated charge-separation energy levels (bottom). a) Tyr-OH…B_A_ and H_A_–CH_3_…Mg^2+^–B_A_ conformation [as originally assigned in the 2.01 Å structure, PDB code: 3I4D (27)]. b) Tyr-OH…P_B_ and H_A_–CH_3_…Mg^2+^–B_A_ conformation. c) Tyr-OH…B_A_ and H_A_–C=O…Mg^2+^–B_A_ conformation. d) Tyr-OH…P_B_ and H_A_–C=O…Mg^2+^–B_A_ conformation [as originally assigned in the 1.87 Å structure, PDB code: 2J8C (28)]. Black labels indicate conformations originally assigned in the 2.01 Å structure, whereas blue labels indicate alternative conformations. Red balls represent the oxygen atoms of methyl-keto groups in P_A_ and H_A_ and tyrosine. Black horizontal bars indicate energy levels of electronically excited states, calculated using the P_A_P_B_ dimer and the B_A_ and B_B_ monomer models, and of charge-separated intermediates, calculated using the P_A_P_B_B_A_H_A_ tetramer model. Black dotted horizontal bars represent energy levels for charge-separated intermediates calculated using the P_A_P_B_B_A_ trimer model for comparison. Note that the lack of a given charge-separated species in either the P_A_P_B_B_A_ trimer or the P_A_P_B_B_A_H_A_ tetramer indicate that the corresponding state is energetically inaccessible in the present conditions (eg [P_A_P_B_]^•**+**^B_A_^•−^, [d]). Arrows indicate major charge-separation pathways deduced in the present results, shown for clarity. For simplicity, these major pathways are defined as comprising (i) the electronically excited site serving as the initial electron donor, (ii) followed by a more stable charge-separated state involving oxidation of the donor, and (iii) direct charge separation between adjacent cofactors (ie between [P_A_P_B_] and B_A_, or between B_A_ and H_A_, not between [P_A_P_B_] and H_A_).

Below, we investigate the charge-separation energetics of all four combinations of P_B_ and H_A_ methyl-keto conformations, using the 2.01-Å structure. While multiple charge-separation pathways may coexist in each conformation, we focus, for simplicity, on the major pathway that is unique to each conformation: these major pathways are defined as comprising (i) the electronically excited site serving as the initial electron donor, (ii) followed by a more stable charge-separated state involving oxidation of the donor, and (iii) direct charge separation between adjacent cofactors (ie between [P_A_P_B_] and B_A_, or between B_A_ and H_A_, not between [P_A_P_B_] and H_A_, see Fig. 2).

**Figure 3:**
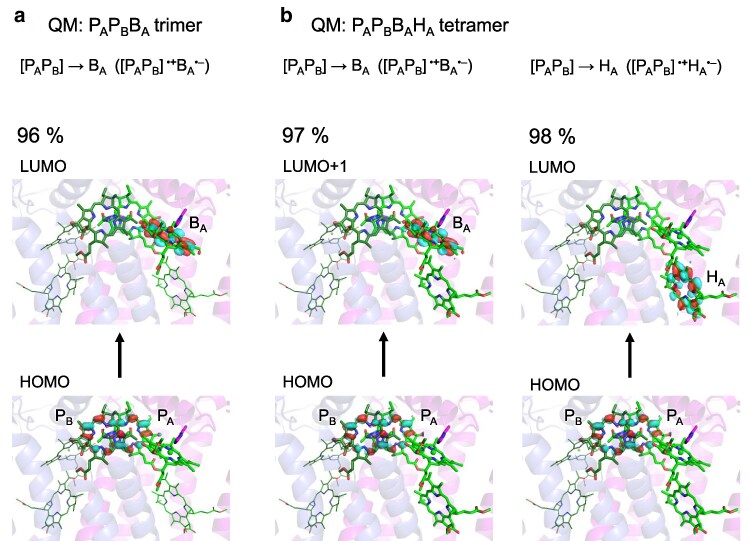
Orbital characteristics of charge-transfer excited states relevant to initial charge separation for the Tyr-OH…B_A_ and H_A_–CH_3_…Mg^2+^–B_A_ conformation (Fig. 2a) in the PbRC protein environment. a) Quantum-chemically treated QM region consisting of the P_A_P_B_B_A_ trimer, showing the donor–acceptor orbital characteristics of the [P_A_P_B_]^•**+**^B_A_^•−^ charge-transfer excitation. b) QM region consisting of the P_A_P_B_B_A_H_A_ tetramer, showing the donor–acceptor orbital characteristics of the [P_A_P_B_]^•**+**^B_A_^•−^ and [P_A_P_B_]^•**+**^H_A_^•−^ charge-transfer excitations. For each excitation, the occupied (the highest occupied molecular orbital, HOMO) and unoccupied (the lowest unoccupied molecular orbital, LUMO, or LUMO + 1) molecular orbitals that dominantly contribute to the excitation are shown. Arrows indicate the orbital transition associated with each excitation. The values (%) indicate the contribution of the depicted orbital transition to the corresponding charge-transfer excitation vector. The clear separation between occupied and unoccupied orbitals confirms the charge-transfer character of these excitations. Oscillator strength for each excitation is listed in Table S2. Orbital representations for all analyzed excitations, including locally excited states (eg [P_A_P_B_]*) and those in other methyl-keto conformations (Fig. 2b–d), are provided in Fig. S2.

In the Tyr-OH…B_A_ conformation of the P_B_ methyl-keto group, the hydroxyl hydrogen of Tyr-M210 is directed toward B_A_ and away from P_B_ (Fig. 2a and c). This Tyr-OH…B_A_ conformation can effectively stabilize the charge-separated [P_A_P_B_]^•**+**^B_A_^•−^ state, as suggested previously (10, 11, 16–18), compared to the Tyr-OH…O=C–P_B_ conformation (Fig. 2b and d). In the Tyr-OH…B_A_/H_A_–CH_3_…Mg^2+^–B_A_ conformation (Fig. 2a), the [P_A_P_B_]^•**+**^H_A_^•−^ state is more stable than the [P_A_P_B_]^•**+**^B_A_^•−^ state and the [P_A_P_B_]^•**+**^B_A_^•−^ state is more stable than the electronically excited [P_A_P_B_]* state, suggesting a conventional charge-separation pathway of [P_A_P_B_]* → [P_A_P_B_]^•**+**^B_A_^•−^ → [P_A_P_B_]^•**+**^H_A_^•−^ (Figs. 2a, 3, and S2).

In contrast, when the P_B_ methyl-keto group reorients and adopts the Tyr-OH…P_B_/H_A_–CH_3_…Mg^2+^–B_A_ conformation, the [P_A_P_B_]^•**+**^B_A_^•−^ state becomes energetically unfavorable, lying even above [P_A_P_B_]* (Fig. 2b), due to destabilization by the orientation of the Tyr-M210 hydroxyl group, whose proton faces [P_A_P_B_]^•**+**^ and oxygen faces B_A_^•−^. Consequently, charge separation via the conventional [P_A_P_B_]* → [P_A_P_B_]^•**+**^B_A_^•−^ pathway is suppressed.

Intriguingly, however, in this conformation, an alternative state, the B_A_^•**+**^H_A_^•−^ state becomes significantly stable, even more so than the [P_A_P_B_]* and B_A_* states. The enhanced stability of the B_A_^•**+**^H_A_^•−^ state arises because the hydroxyl oxygen of Tyr-M210 faces B_A_^•+^ and stabilizes it electrostatically (Fig. 2b). Thus, the Tyr-OH…P_B_ conformation appears to facilitate charge separation via the alternative B_A_* to B_A_^•**+**^H_A_^•−^ pathway, rather than the conventional [P_A_P_B_]* → [P_A_P_B_]^•**+**^B_A_^•−^ pathway.

Notably, even in the Tyr-OH…B_A_ conformation of P_B_, the alternative B_A_* → B_A_^•**+**^H_A_^•−^ pathway is favored if H_A_ adopts the H_A_–C=O…Mg^2+^–B_A_ conformation (Fig. 2c). In this case, stabilization of the B_A_^•+^H_A_^•−^ state results from: (i) the carbonyl oxygen of the H_A_ methyl-keto group coordinates axially to the Mg^2+^ of B_A_, effectively stabilizing the B_A_^•+^ species through an axial-ligand-like interaction; simultaneously (ii) the anionic H_A_^•−^ species is also stabilized by the presence of cationic Mg^2+^ of B_A_.

Overall, except for the Tyr-OH…B_A_/H_A_–CH_3_…Mg^2+^–B_A_ conformation, which facilitates the conventional [P_A_P_B_]* → [P_A_P_B_]^•**+**^B_A_^•−^ charge separation (Fig. 2a), all other conformations (Fig. 2b–d) favors the B_A_^•**+**^H_A_^•−^ state over the [P_A_P_B_]^•**+**^B_A_^•−^ state (Fig. 2b–d). The involvement of the B_A_^•**+**^H_A_^•−^ state in charge separation resembles the Chl_D1_^•**+**^Pheo_D1_^•−^ state in charge separation of PSII (8).

Notably, direct evidence for the B_A_*-initiated pathway has been reported at low temperatures (31–33). Therefore, the Tyr-OH…P_B_ conformation of P_B_ and the H_A_–C=O…Mg^2+^–B_A_ conformation of H_A_ enables this alternative B_A_* → B_A_^•**+**^H_A_^•−^ charge separation pathway. These results suggest that these methyl-keto conformations differentiate the charge separation mechanisms drastically, either involving the [P_A_P_B_]^•**+**^B_A_^•−^ or B_A_^•**+**^H_A_^•−^ states.

## Discussion

### P_B_ methyl-keto orientations

**Figure 4:**
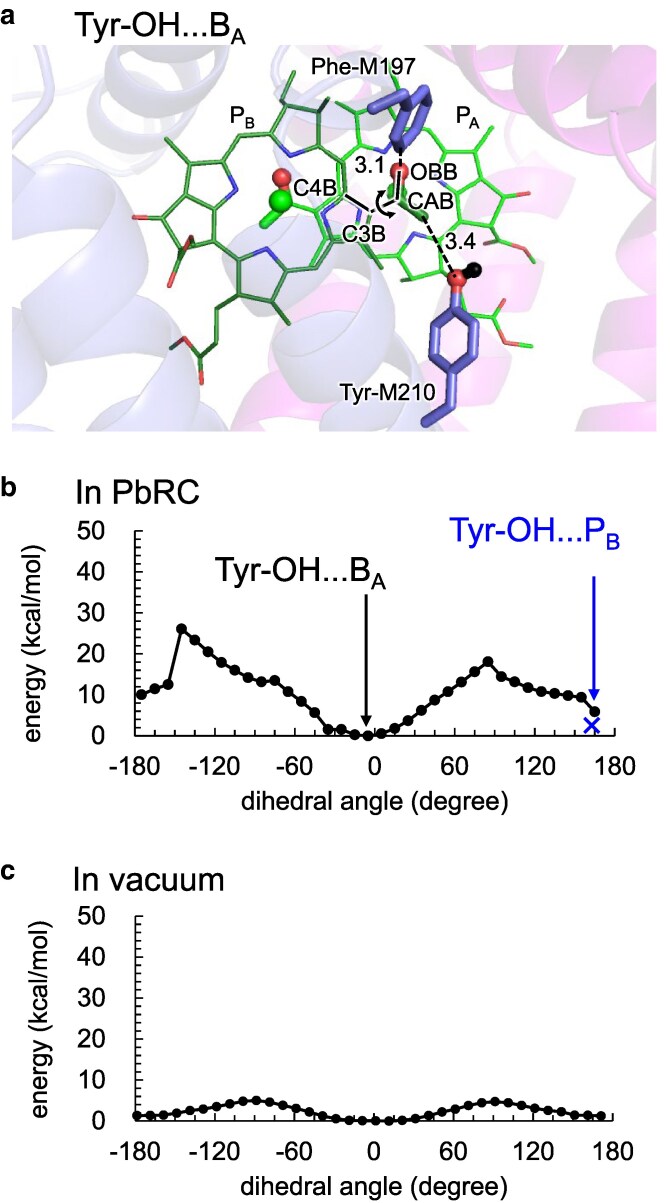
Kinetically isolated methyl-keto conformations of P_B_. a) Definition of the methyl-keto dihedral angle in P_B_ (OBB…CAB…C3B…C4B). The curved arrow indicates the direction of rotation. b) Potential energy profile for rotation of the methyl-keto group in P_B_ in the presence of the PbRC protein environment. The profile starts from the Tyr-OH…B_A_ conformation at the global minimum around 0° (closed circles). A local minimum is observed around ±180°, where the hydroxyl oxygen of Tyr-M210 is within H-bonding distance of the P_B_ methyl-keto group but no H-bond is formed in the QM/MM-optimized geometry. For reference, a structure obtained by reorienting the hydroxyl hydrogen toward the P_B_ methyl-keto group and subsequently performing QM/MM optimization, corresponding to the Tyr-OH…P_B_ conformation but differing slightly from the one used in Fig. 2b, is also shown (cross). c) Potential energy profile for rotation of the methyl-keto group in P_B_ in vacuum (in the absence of the PbRC protein environment). The potential energy profiles were obtained from fully relaxed QM/MM dihedral scans. The QM region consisted of P_A_P_B_B_A_ and their axial histidine ligands. The dihedral angle (OBB…CAB…C3B…C4B) was incrementally rotated by 10°, and at each step, geometry optimization was performed at the B3LYP/LACVP* level with the dihedral angle constrained. In the MM region, H atoms were optimized, whereas heavy atoms were kept fixed except for the side chains of Val-L157, Phe-M197, and Tyr-M210 near the P_B_ methyl-keto group.

In the absence of the PbRC protein environment, the two methyl-keto conformations of P_B_ are nearly isoenergetic and readily interconvert due to low rotational barriers (25) (Fig. 4c). In contrast, within the PbRC protein matrix, the Tyr-OH…B_A_ conformation is energetically more stable than the Tyr-OH…P_B_ conformation (Fig. 4b). This stabilization originates from specific local nonbonded interactions at the P_B_ binding site. In particular, the polar oxygen atom of the P_B_ methyl-keto group is positioned closer to the Mg^2+^ site of P_A_ in the Tyr-OH…B_A_ conformation (3.6 Å) than in the Tyr-OH…P_B_ conformation (4.2 Å) in the QM/MM-optimized geometries (Supporting Datasets), which may contribute to more favorable local electrostatic stabilization in the former within the π-stacked [P_A_P_B_] dimer. Thus, although the overall protonation pattern of the protein remains unchanged between the two conformations, orientation-dependent local electrostatic interactions at the P_B_ binding site, arising from the different polarity of the methyl and keto moieties, substantially modulate the relative energetics.

In addition, [P_A_P_B_]* is energetically lower than B_A_*. These factors together could explain why the [P_A_P_B_]* → [P_A_P_B_]^•**+**^B_A_^•−^ → [P_A_P_B_]^•**+**^H_A_^•−^ charge separation (Fig. 2a) has been more commonly observed as a canonical charge separation, compared to B_A_* → B_A_^•**+**^H_A_^•−^ (Fig. 2b). Consequently, under thermodynamic equilibrium at physiological temperatures, the Tyr-OH…B_A_ conformation should be a dominant species.

This interpretation is consistent with the previous analysis as follows (17): while in the 2.01 Å structure (PDB code: 3I4D) (27), the P_B_ methyl-keto orientation adopts the Tyr-OH…B_A_ conformation, the 1.87 Å structure (PDB code: 2J8C) (28)) assigns the P_B_ methyl-keto group to the Tyr-OH…P_B_ conformation (Fig. 1). However, the electron density observed in the 1.87 Å structure is not consistent with this assignment; only when the Tyr-OH…B_A_ conformation is modeled does the original density align unambiguously.

The closest groups to the P_B_ methyl-keto moiety are Phe-M197 (3.1 Å) and Tyr-M210 (3.4 Å), both bulky aromatic side chains. The two rotational barriers located at +85° and −145° (Fig. 4b) arise primarily from steric interactions with Mg^2+^ in P_A_ and a carbon atom (CMD) in B_A_, respectively, each of which approaches the P_B_ methyl-keto group at van der Waals contact distances (Fig. S3). Although molecular dynamics simulations including these cofactors (34, 35) would allow local structural relaxation and likely reduce the apparent barrier, the inherent proximity of Mg^2+^ in P_A_ and the chlorin ring of B_A_ imposes unavoidable packing constraints in the protein environment. Thus, once the P_B_ methyl-keto orientation is established during protein folding and cofactor assembly, substantial reorientation within the assembled PbRC is expected to be energetically disfavored.

### H_A_ methyl-keto orientations

**Figure 5:**
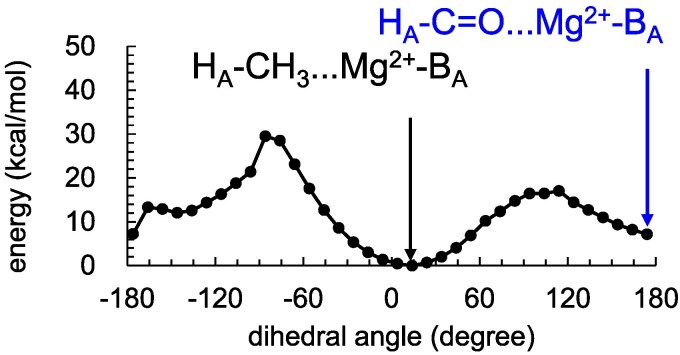
Potential energy profile for rotation of the methyl-keto group in H_A_ for the Tyr-OH…B_A_ conformation. The H_A_–CH_3_…Mg^2+^–B_A_ conformation corresponds to the global energy minimum around 0°, whereas the H_A_–C=O…Mg^2+^–B_A_ conformation corresponds to a local energy minimum around 180°. The potential energy profiles were obtained from fully relaxed QM/MM dihedral scans. The QM region consisted of B_A_H_A_. The dihedral angle (OBB…CAB…C3B…C4B) was incrementally rotated by 10°, and at each step, geometry optimization was performed at the B3LYP/LACVP* level with the dihedral angle constrained. In the MM region, the positions of H atoms were optimized, while the positions of the heavy atoms were kept fixed except for the side chains of Tyr-M210 and the phytyl chain (C2 to C20) of P_A_ near the H_A_ methyl-keto group.

The H_A_–CH_3_…Mg^2+^–B_A_ conformation is energetically more stable than H_A_–C=O…Mg^2+^–B_A_ conformation (Fig. 5). The rotational barriers at +114° and −86° appear to originate from steric interactions with the ether oxygen (O2A) in the B_A_ phytyl ester group and a carbon atom (C5) in the P_A_ phytyl chain (Fig. S3).

Considering the H_A_ methyl-keto energetics together with the P_B_ methyl-keto conformations, where the Tyr-OH…B_A_ conformation is more stable than the Tyr-OH…P_B_ conformation (Fig. 4), the methyl-keto conformations are likely populated in the following order: [Tyr-OH…B_A_/H_A_–CH_3_…Mg^2+^–B_A_] (Fig. 2a) > [Tyr-OH…B_A_/H_A_–C=O…Mg^2+^–B_A_] (Fig. 2c) ≈ [Tyr-OH…P_B_/H_A_–CH_3_…Mg^2+^–B_A_] (Fig. 2b) > [Tyr-OH…P_B_/H_A_–C=O…Mg^2+^–B_A_] (Fig. 2d). Thus, as the most populated conformation is [Tyr-OH…B_A_/H_A_–CH_3_…Mg^2+^–B_A_], the canonical charge separation in PbRC proceeds via [P_A_P_B_]* → [P_A_P_B_]^•**+**^B_A_^•−^ → [P_A_P_B_]^•**+**^H_A_^•−^ (Fig. 2a), even though the other three conformations allow the alternative B_A_* → B_A_^•**+**^H_A_^•−^ charge separation pathway.

While the H_A_–CH_3_…Mg^2+^–B_A_ conformation likely represents the physiologically dominant form under ambient conditions, it seems plausible that the alternative H_A_–C=O…Mg^2+^–B_A_ conformation can still exist. In particular, at low temperatures, less stable conformations may become kinetically trapped. This could explain why the alternative B_A_* to B_A_^•**+**^H_A_^•−^ electron transfer is predominantly observed at low temperatures (31–33).

Spectroscopic studies by Müh et al. have suggested that the initial H_A_^•−^ state (“I_(1)_^•−^” state (23)) becomes further stabilized upon reorientation of the H_A_-methyl-keto group, forming a more stable H_A_^•−^ state (“I_(2)_^•−^” state (23)) in the Q_A_-depleted PbRC. According to the present results, these observations can be interpreted as follows: the initial H_A_^•−^ state, I_(1)_^•−^, forms in the Tyr-OH…B_A_/H_A_–CH_3_…Mg^2+^–B_A_ conformation (Fig. 2a). The *apparent* energy barrier for H_A_ methyl-keto reorientation shown in Fig. 5, obtained in the absence of protein dynamics, suggests that such reorientation requires protein fluctuations to overcome steric constraints. For example, the Tyr-M210 side chain is located at van der Waals contact distance (3.4 Å) from the H_A_ methyl-keto group, and its fluctuation is expected to accompany rotation of the H_A_ methyl-keto group. Consistent with this interpretation, H_A_ methyl-keto reorientation was observed by Müh et al. in the absence of Q_A_ (23), where the prolonged lifetime of the H_A_^•−^ state allows sufficient time for protein dynamics to facilitate methyl-keto reorientation.

Upon inhibition of electron transfer from I_(1)_^•−^ to Q_A_, the H_A_ methyl-keto reorients, and I_(2)_^•−^ forms in the H_A_–C=O…Mg^2+^–B_A_ conformation (Fig. 2c). Crucially, had this reorientation occurred prior to excitation, charge separation via the [P_A_P_B_]^•**+**^H_A_^•−^ state would have been energetically unfavorable, because the [P_A_P_B_]^•**+**^B_A_^•−^ state is significantly destabilized in the reoriented, H_A_–C=O…Mg^2+^–B_A_ conformation. Thus, the sequence of events is critical: the H_A_ methyl-keto reorientation must follow, not precede, the formation of the [P_A_P_B_]^•**+**^H_A_^•−^ state.

Overall, the H_A_–C=O…Mg^2+^–B_A_ conformation leads to further stabilization of the H_A_^•−^-containing state, by upshifting *E*_m_(H_A_). However, it simultaneously destabilizes the canonical charge-separation species, [P_A_P_B_]^•**+**^B_A_^•−^. These suggest that the canonical charge-separation proceeds via the H_A_–CH_3_…Mg^2+^–B_A_ conformation. Once the [P_A_P_B_]^•**+**^H_A_^•−^ state forms and further electron transfer is inhibited [eg in the absence of Q_A_ (23)], it can subsequently be stabilized by reorientation of the H_A_ methyl-keto group into the H_A_–C=O…Mg^2+^–B_A_ conformation. Thus, the H_A_ methyl-keto reorientation likely functions as a postcharge-separation stabilization mechanism rather than as a facilitator of initial charge separation process.

The alternative B_A_* → B_A_^•**+**^H_A_^•−^ charge separation pathway in PbRC resembles the charge separation pathway in PSII, where Chl_D1_*-initiated charge separation operates. In PSII, this mechanism ultimately originates from the electrostatic protein environment generated by its water-splitting apparatus, including the oxygen-evolving Mn_4_CaO_5_ cluster and associated proton-transfer pathways, whose negative charges on the D1 side stabilizes the nearby Chl_D1_^•+^ state (eg theoretical studies (8, 36) and 2D electronic spectroscopic studies (37, 38)). In contrast, the alternative B_A_* → B_A_^•**+**^H_A_^•−^ charge separation pathway in PbRC arises from specific orientations of the polar methyl-keto groups in the chlorin cofactors. While these two systems rely on distinct molecular mechanisms, electrostatic stabilization in PSII vs. conformational control in PbRC, the results presented here indicate that the functionality of the accessory chlorophyll as an initial electron donor, canonical in PSII, is not absent in PbRC, but rather implicitly conserved and can be functionally accessed by reorienting the methyl-keto group, given the common evolutionary origin of PbRC and PSII from an ancestral reaction center (39). This subtle yet effective tuning mechanism in PbRC, mediated by polar residues and substituents, likely reflects an ancestral adaptation. Following the emergence of PSII, the electron-withdrawing nature of the acetyl group (ie methyl-keto group) would remain beneficial for PbRC, making bacteriochlorophylls such as bacteriochlorophyll *a* more resistant to oxidation than their vinyl-containing analogs, eg bacteriochlorophyll *g* (40). In contrast, PSII, which originally evolved with the chemically more stable chlorin macrocycle (eg chlorophyll *a*) may not have required additional stabilization via acetylation, allowing the retention of the vinyl group under oxygenic conditions.

## Conclusions

The present study reveals that the methyl-keto group orientations of bacteriochlorophyll and bacteriopheophytin cofactors in PbRC play a crucial role in modulating the charge-separation pathways and the stability of the resulting electron-transfer intermediates. Two kinetically isolated conformations of the P_B_ methyl-keto group, the Tyr-OH…B_A_ (Fig. 2a) and Tyr-OH…P_B_ (Fig. 2b), determine the hydroxyl orientation of the key polar residue Tyr-M210 near P_B_ and B_A_ (10, 11, 16–18), thereby modulating the electrostatic stabilization of charge-separated intermediates. As a consequence, PbRC can access either the canonical [P_A_P_B_]* → [P_A_P_B_]^•+^B_A_^•−^ → [P_A_P_B_]^•+^H_A_^•−^ pathway (Fig. 2a) or an alternative B_A_* → B_A_^•+^H_A_^•−^ pathway (Fig. 2b). In addition, reorientation of the H_A_ methyl-keto group from the H_A_–CH_3_…Mg^2+^–B_A_ conformation to the H_A_–C=O…Mg^2+^–B_A_ conformation further stabilizes the B_A_^•+^H_A_^•−^ state, expanding the accessibility of the alternative B_A_* → B_A_^•+^H_A_^•−^ pathway (Fig. 2c and d). Overall, the present results provide a molecular-level rationale for the coexistence of multiple charge-separation pathways in PbRC.

## Methods

### Coordinates and atomic partial charges

The initial atomic coordinates were obtained from the 2.01 Å crystal structure of *Rhodobacter sphaeroides* PbRC (PDB code: 3I4D) (27). For comparison, the present study also refers to another available high-resolution structure: the 1.87 Å structure (PDB code: 2J8C) (28). To maintain the consistency, the side chain orientations of two asparagine residues near P_A_P_B_, in the 2.01 Å structure, specifically Asn-L159 and Asn-M187, were tentatively adopted from those in the 1.87 Å structure (Fig. S4), because the positions of the side chain N and O atoms in these residues cannot be unambiguously determined from the electron density alone, even at 2.01 Å resolution.

Atomic partial charges of amino acids were derived from the all-atom CHARMM22 (41) parameter set. Atomic charges for PbRC cofactors, including bacteriochlorophyll *a*, bacteriopheophytin *a*, the nonheme Fe complex, ubiquinone, and spheroidene were adopted from previous studies (17), in which the charges were determined by fitting the electrostatic potential around these molecules using the RESP procedure (42). The electronic wave functions were obtained after geometry optimization using the density functional theory (DFT) method (B3LYP/LACVP*) in JAGUAR (43). For the atomic charges of the nonpolar CH_n_ groups in cofactors (eg the phytol chains of bacteriochlorophyll and bacteriopheophytin and the isoprene side chains of quinones), a charge of +0.09 was assigned to the nonpolar hydrogen atoms, following the CHARMM framework (41, 44). The positions of all heavy atoms were fixed, and all titratable groups, such as acidic and basic groups, were ionized during optimization of H-atom positions using CHARMM (45). Ligand histidine residues were maintained in charge-neutral, singly protonated states. All other acidic and basic groups were ionized.

Atomic partial charges are predominantly used to generate initial coordinates and to define the electrostatic embedding in the MM region. Because the electron-transfer-active cofactors are explicitly treated in the QM region, the atomic charges of the cofactors themselves have minimal influence on the results. Instead, the surrounding amino acid residues, represented in the MM region, define the electrostatic environment that interacts with the QM region. The amino acid charges used in this study are from the standard CHARMM22 all-atom force field, which has been widely adopted for decades and whose charges for standard residues have remained essentially unchanged among CHARMM versions. Importantly, the cofactors in PbRC are located in the hydrophobic transmembrane region, where no bulk water is present.

As noted, the charge set for cofactors used in this study was derived from DFT calculations and fitted to the electrostatic potential using the RESP procedure (42). While alternative charge sets, with essentially similar values, have also been applied to PbRC in other theoretical studies (46, 47), the charge set employed in the present work has, to our knowledge, been most extensively applied and validated. Specifically, it has been shown to successfully reproduce experimentally measured values associated with electrostatics such as redox potentials, p*K*_a_ values, and energetics in the charge-separated states. To our knowledge, no alternative charge set has been reported to outperform this charge set in reproducing such experimentally measured values in photosynthetic reaction centers. Therefore, for consistency and comparability with our previous studies, we retain this charge set in the present work.

### Protonation pattern

The protonation states of titratable residues were calculated by solving the linear Poisson–Boltzmann equation using the MEAD program (48). To ensure consistency with previous computational results (17, 49), all calculations were performed at 300 K, pH 7.0, and an ionic strength of 100 mM. Dielectric constants of 4 (protein interior) and 80 (bulk water) were used. For p*K*_a_ calculations, crystal water molecules were removed and replaced with a dielectric continuum (dielectric constant: 80), to avoid artificial fixation of H-bond patterns due to H-atom orientations in the initial atomic coordinates of explicit water molecule, which could bias the protonation states of nearby residues.

p*K*_a_ values of titratable sites in the protein were determined by adding the calculated p*K*_a_ shift relative to a reference system to the known reference p*K*_a_ values: 12.0 for Arg, 4.0 for Asp, 9.5 for Cys, 4.4 for Glu, 10.4 for Lys, 9.6 for Tyr (50), and 7.0 and 6.6 for the N_ε_ and N_δ_ atoms of His, respectively (51–53). The linear Poisson–Boltzmann equation was solved through a three-step grid-focusing procedure at resolutions of 2.5, 1.0, and 0.3 Å. During each titration step, all other titratable sites were fully equilibrated to the protonation state of the target site during titration, which was performed using Monte Carlo sampling as implemented in Karlsberg (54). The probabilities of the protonated and deprotonated states obtained from the sampling were used to evaluate the p*K*_a_ values via the Henderson–Hasselbalch equation. A bias potential was applied to equalize the two states ([protonated] = [deprotonated]), and the corresponding potential was taken as the p*K*_a_ value. The resulting protonation patterns were subsequently used in the QM/MM calculations.

The protonation patterns of titratable residues were calculated independently for each methyl-keto conformation. However, the overall protonation pattern of the protein remained unchanged between the two conformations. The closest acidic or basic residue to the P_B_ methyl-keto group is Asp-L155, located on the periplasmic side. The distance between the side-chain oxygen of Asp-L155 and the methyl-keto oxygen of P_B_ is ∼11 Å for the outward-facing conformation and ∼13 Å for the inward-facing conformation. Therefore, the orientation of this charge-neutral methyl-keto group did not affect the protonation state of Asp-L155 or any other titratable residue.

### QM/MM calculations

The unrestricted DFT method was employed using the B3LYP functional and LACVP* basis sets, as implemented in the QSite (55) program. For geometry optimization, the QM region was defined as follows: P_A_P_B_ was treated as a bacteriochlorophyll dimer with axial histidine ligands; B_A_ and B_B_ were treated as individual bacteriochlorophyll monomers with histidine ligands; and H_A_ and H_B_ were treated as individual bacteriopheophytin monomer. The phytol chains, composed of nonpolar CH_n_ groups, were included in the MM region. See Supporting Datasets for the QM/MM-optimized atomic coordinates. All atomic coordinates were fully relaxed in the QM region. In the MM region, the positions of H atoms were optimized using the OPLS2005 force field (56), while the positions of the heavy atoms were fixed. The MM region also included polar side chains and water molecules participating in the same H-bond network near P_A_P_B_, specifically Ser-L158, Asn-L159, Tyr-L162, Asn-M187, Ser-M190, and Asn-M195 (Fig. S4), which were preoptimized using the CHARMM force field prior to the QM/MM calculations.

### Potential energy profile

To analyze the potential energy profiles associated with the methyl-keto rotations in P_B_ and H_A_, the QM regions were defined as P_A_P_B_B_A_ with their axial histidine ligands and B_A_H_A_, respectively. The QM/MM optimized geometry was used as the starting structure. The dihedral angle (OBB…CAB…C3B…C4B) was incrementally rotated by 10°, and at each step, geometry optimization was performed with the dihedral angle constrained. In the MM region, heavy atoms were kept fixed, while hydrogen atoms were optimized. For the P_B_ methyl-keto rotation, the side chains of Val-L157, Phe-M197, and Tyr-M210 were also optimized. For the H_A_ methyl-keto rotation, the side chain of Tyr-M210 and the phytyl chain (C2–C20 atoms) of P_A_ were optimized. This procedure was repeated in both rotational directions until the dihedral angle reached −180° and 180°.

### Excited states and charge-separated states

To calculate excited states, we employed a QM/MM approach combined with the polarizable continuum model (PCM) method using a dielectric constant of 80. The QuanPol method (57), implemented in the GAMESS program (58), was used to describe interactions between QM and MM atoms. This method explicitly accounts for the electrostatic and steric effects of the protein environment in the presence of bulk water. In the PCM method, polarization points were placed on spheres with a radius of 3.0 Å centered on each atom.

To analyze the energetics of electronically excited states and charge separated states (ie donor–acceptor charge-transfer excited states), the time-dependent density functional theory (TDDFT) was employed with the CAMB3LYP functional (59), where the range-separation parameters *μ* of 0.14 (60), *α* of 0.19, and *β* of 0.46 were used. The QM regions were defined as follows: the B_A_ monomer for the electronically excited B_A_* state, the B_B_ monomer for the B_B_* state, the P_A_P_B_ dimer for the [P_A_P_B_]* state, and the P_A_P_B_B_A_ trimer or the P_A_P_B_B_A_H_A_ tetramer for the charge-separated [P_A_P_B_]^•**+**^B_A_^•−^, [P_A_P_B_]^•**+**^H_A_^•−^, and B_A_^•**+**^H_A_^•−^ states, as previously analyzed in studies of charge separation in PbRC and PSII (8). Within this framework, TDDFT provides vertical excitation energies from the neutral ground state, including donor–acceptor charge-transfer excitations involving single-electron transfer between cofactors. Low-oscillator-strength excited states exhibiting clear donor–acceptor orbital localization are interpreted as charge-transfer (charge-separated) states (eg [P_A_P_B_]^•+^B_A_^•−^, [P_A_P_B_]^•+^H_A_^•−^, and B_A_^•+^H_A_^•−^ states), whereas states with significant oscillator strength and localized orbital character are assigned as locally excited states (Table S2). These excited-state configurations are accessible via TDDFT, provided that long-range exchange is appropriately treated, which is the primary reason for employing CAM-B3LYP. The number of excited states analyzed was limited by the large size of the QM region and the associated memory demands of TDDFT calculations. Accordingly, only the lowest-energy excited states, up to eight states for the P_A_P_B_B_A_ trimer and up to four states for the P_A_P_B_B_A_H_A_ tetramer, were analyzed under the present computational conditions. Therefore, complementary P_A_P_B_B_A_ trimer calculations are included to enable analysis of a broader set of low-energy excitations in addition to P_A_P_B_B_A_H_A_ tetramer calculations.

The primary charge-separation occurs on the picosecond timescale in PbRC (20), a process that is widely treated as a vertical (ie nonequilibrated) excitation. On such a timescale, and large-scale protein rearrangements and slow dielectric/structural relaxation are not expected to fully equilibrate with the newly formed charge distribution during the event. Consistent with this picture, time-resolved X-ray free-electron laser structures of PbRC reported only subtle differences in early intermediates (61), and subsequent analyses argued that even the observed structural changes are at the limit of the experimental resolution and were not associated with redox changes in the cofactors (49). Given these, molecular dynamics simulations were not conducted for each charge-separated state, and the present QM/MM calculations are expected to sufficiently approximate the electrostatic environment relevant to primary charge separation, as commonly assumed in theoretical treatments of ultrafast reaction-center charge separation (62, 63).

## Supplementary Material

pgag111_Supplementary_Data

## Data Availability

All data are included in the manuscript and/or Supporting information.
