# Supplementary material for: Molecular basis for alternative charge separation pathways in type-II photosynthetic reaction centers
Source: PNAS Nexus. 2026 Apr 9;5(4):pgag111. doi: 10.1093/pnasnexus/pgag111 (PMC13112432; doi:10.1093/pnasnexus/pgag111)
Supplement: pgag111_Supplementary_Data [file pgag111_supplementary_data.zip › PbRC-cation-SI_4.1_HI_unmarked.pdf]

Supporting Information

Molecular basis for alternative charge separation pathways in type-II photosynthetic reaction centers

Tomoyasu Noji [1,2], Keisuke Saito [1,2], Hiroyuki Tamura [1,2], and Hiroshi Ishikita [1,2]*

1) Department of Applied Chemistry, The University of Tokyo, 7-3-1 Hongo, Bunkyo-ku, Tokyo 113-8654, Japan

2) Research Center for Advanced Science and Technology, The University of Tokyo, 4-6-1 Komaba, Meguro-ku, Tokyo 153-8904, Japan

CORRESPONDING AUTHOR: Ishikita, Research Center for Advanced Science and Technology, The University of Tokyo, 4-6-1 Komaba, Meguro-ku, Tokyo 153-8904, Japan, Tel. +81-3-5452-5056, Fax. +81-3-5452-5083, **E-mail:** hiro@appchem.t.u-tokyo.ac.jp

Contents

12 pages

2 tables

4 figures

Table S1. Conformational differences in the P_B and H_A methyl-keto the orientations.

**PDB code**	**resolution**	**P_B methyl-keto**	**H_A methyl-keto**
3I4D	2.01	Tyr-OH...B_A	H_A-CH_3...Mg^{2+}-B_A
2J8C	1.87	Tyr-OH...P_B	H_A-C=O...Mg^{2+}-B_A
2BNP	2.70	Tyr-OH...B_A	H_A-CH_3...Mg^{2+}-B_A
6Z02	2.10	Tyr-OH...P_B	H_A-CH_3...Mg^{2+}-B_A
1PCR	2.65	Tyr-OH...B_A	H_A-C=O...Mg^{2+}-B_A
1KBY	2.50	Tyr-OH...P_B	H_A-C=O...Mg^{2+}-B_A

Table S2. TDDFT results for low-lying electronic excitations relevant to charge separation. (a) Tyr-OH...B_A and H_A-CH_3...Mg^{2+}-B_A conformation. (b) Tyr-OH...P_B and H_A-CH_3...Mg^{2+}-B_A conformation. (c) Tyr-OH...B_A and H_A-C=O...Mg^{2+}-B_A conformation. (d) Tyr-OH...P_B and H_A-C=O...Mg^{2+}-B_A conformation. Listed are the QM region, the dominant donor → acceptor electronic excitation, excitation energies (eV), and oscillator strengths. The electronic configuration indicated in parentheses denotes the physical interpretation of the excitation, i.e., a locally excited state or a charge-transfer (charge-separated) state. The QM region includes the axial histidine ligands of the bacteriochlorophyll cofactors.

The number of electronic excitations analyzed was limited by the large size of the QM region and the associated memory demands of TDDFT calculations. Accordingly, only the lowest-energy excited states were analyzed (up to eight states for the $P_AP_BB_A$ trimer and up to four states for the $P_AP_BB_AH_A$ tetramer).

n.d., not determined because the corresponding excitation does not appear within the analyzed low-energy window.

	QM region	donor → acceptor excitation (resulting configuration)	excitation energy	oscillator strength
(a)	B_B	$B_B \rightarrow B_B$ (B_B*)	1.792	0.3986
	B_A	$B_A \rightarrow B_A$ (B_A*)	1.788	0.3852
	P_A, P_B	$[P_AP_B] \rightarrow [P_AP_B]$ ($[P_AP_B]$*)	1.553	0.5564
	P_A, P_B, B_A	$[P_AP_B] \rightarrow B_A$ ($[P_AP_B]^{\bullet+}B_A^{\bullet-}$)	1.511	0.0068
	P_A, P_B, B_A, H_A	$[P_AP_B] \rightarrow B_A$ ($[P_AP_B]^{\bullet+}B_A^{\bullet-}$)	1.416	0.0002
	P_A, P_B, B_A, H_A	$[P_AP_B] \rightarrow H_A$ ($[P_AP_B]^{\bullet+}H_A^{\bullet-}$)	1.102	0.0000
	P_A, P_B, B_A, H_A	$B_A \rightarrow H_A$ ($B_A^{\bullet+}H_A^{\bullet-}$)	n.d.	n.d.
(b)	B_B	$B_B \rightarrow B_B$ (B_B*)	1.791	0.3990
	B_A	$B_A \rightarrow B_A$ (B_A*)	1.790	0.3820
	P_A, P_B	$[P_AP_B] \rightarrow [P_AP_B]$ ($[P_AP_B]$*)	1.585	0.5265
	P_A, P_B, B_A	$[P_AP_B] \rightarrow B_A$ ($[P_AP_B]^{\bullet+}B_A^{\bullet-}$)	2.070	0.0000
	P_A, P_B, B_A, H_A	$[P_AP_B] \rightarrow B_A$ ($[P_AP_B]^{\bullet+}B_A^{\bullet-}$)	n.d.	n.d.
	P_A, P_B, B_A, H_A	$[P_AP_B] \rightarrow H_A$ ($[P_AP_B]^{\bullet+}H_A^{\bullet-}$)	1.392	0.0000
	P_A, P_B, B_A, H_A	$B_A \rightarrow H_A$ ($B_A^{\bullet+}H_A^{\bullet-}$)	1.241	0.0074
(c)	B_B	$B_B \rightarrow B_B$ (B_B*)	1.792	0.3987
	B_A	$B_A \rightarrow B_A$ (B_A*)	1.788	0.3891

	P_A, P_B	$[P_AP_B] \rightarrow [P_AP_B]$ ($[P_AP_B]^*$)	1.553	0.5558
	P_A, P_B, B_A	$[P_AP_B] \rightarrow B_A$ ($[P_AP_B]^{\bullet+}B_A^{\bullet-}$)	1.696	0.0003
	P_A, P_B, B_A, H_A	$[P_AP_B] \rightarrow B_A$ ($[P_AP_B]^{\bullet+}B_A^{\bullet-}$)	n.d.	n.d.
	P_A, P_B, B_A, H_A	$[P_AP_B] \rightarrow H_A$ ($[P_AP_B]^{\bullet+}H_A^{\bullet-}$)	1.033	0.0000
	P_A, P_B, B_A, H_A	$B_A \rightarrow H_A$ ($B_A^{\bullet+}H_A^{\bullet-}$)	1.231	0.0023
(d)	B_B	$B_B \rightarrow B_B$ (B_B^*)	1.791	0.3990
	B_A	$B_A \rightarrow B_A$ (B_A^*)	1.788	0.3865
	P_A, P_B	$[P_AP_B] \rightarrow [P_AP_B]$ ($[P_AP_B]^*$)	1.582	0.5152
	P_A, P_B, B_A	$[P_AP_B] \rightarrow B_A$ ($[P_AP_B]^{\bullet+}B_A^{\bullet-}$)	n.d.	n.d.
	P_A, P_B, B_A, H_A	$[P_AP_B] \rightarrow B_A$ ($[P_AP_B]^{\bullet+}B_A^{\bullet-}$)	n.d.	n.d.
	P_A, P_B, B_A, H_A	$[P_AP_B] \rightarrow H_A$ ($[P_AP_B]^{\bullet+}H_A^{\bullet-}$)	1.321	0.0000
	P_A, P_B, B_A, H_A	$B_A \rightarrow H_A$ ($B_A^{\bullet+}H_A^{\bullet-}$)	0.973	0.0002

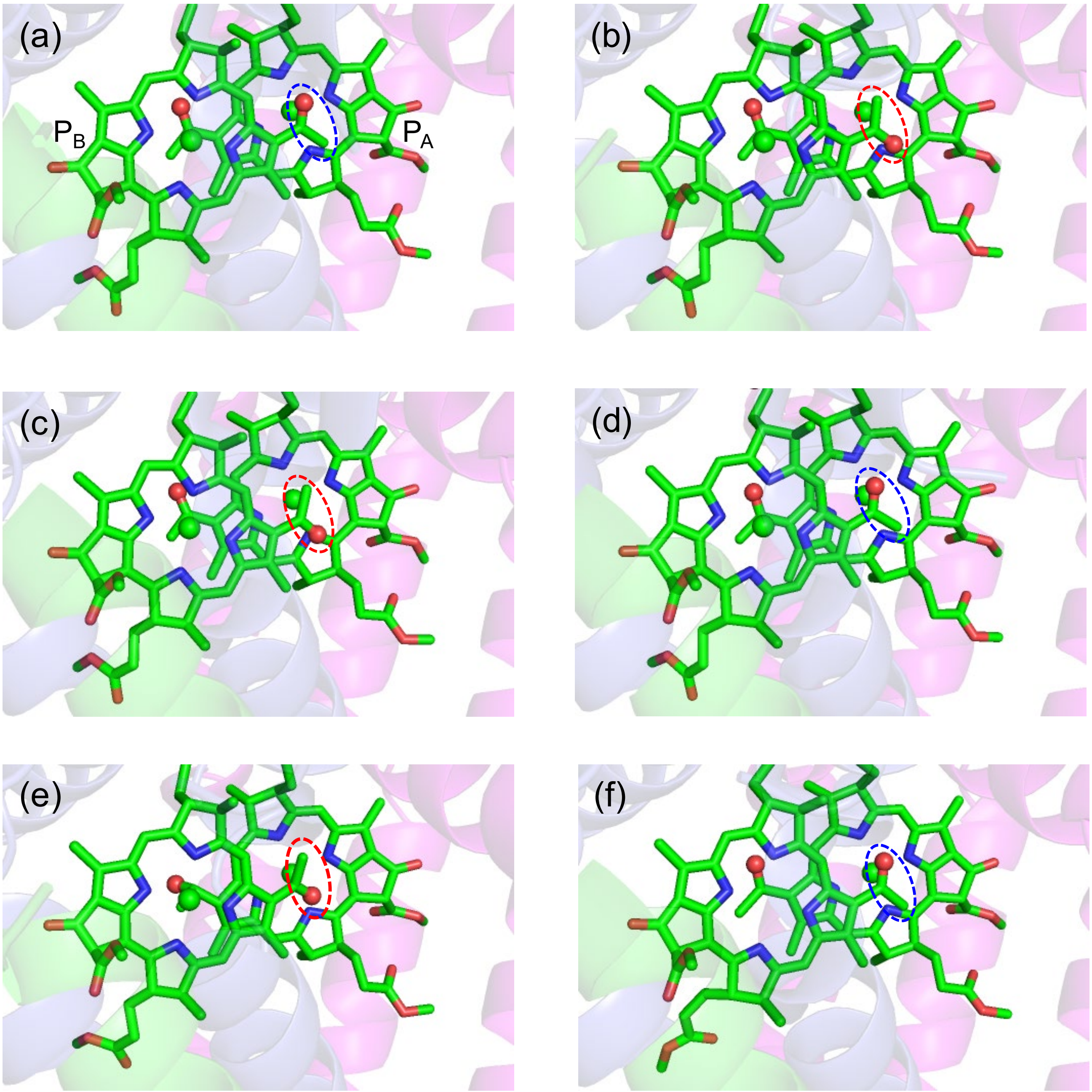

Figure S1. Conformational differences in the orientation of the P_B methyl-keto groups in reported PbRC crystal structures. Structures shown are from the following PDB entries: (a) 3I4D; (b) 2J8C; (c) 2BNP; (d) 6Z02; (e) 1PCR; (f) 1KBY. Red ovals indicate methyl-keto conformations matching that in the 2.01 Å structure (PDB code: 3I4D), whereas blue ovals indicate orientations matching that in the 1.87 Å structure (PDB code: 2J8C).

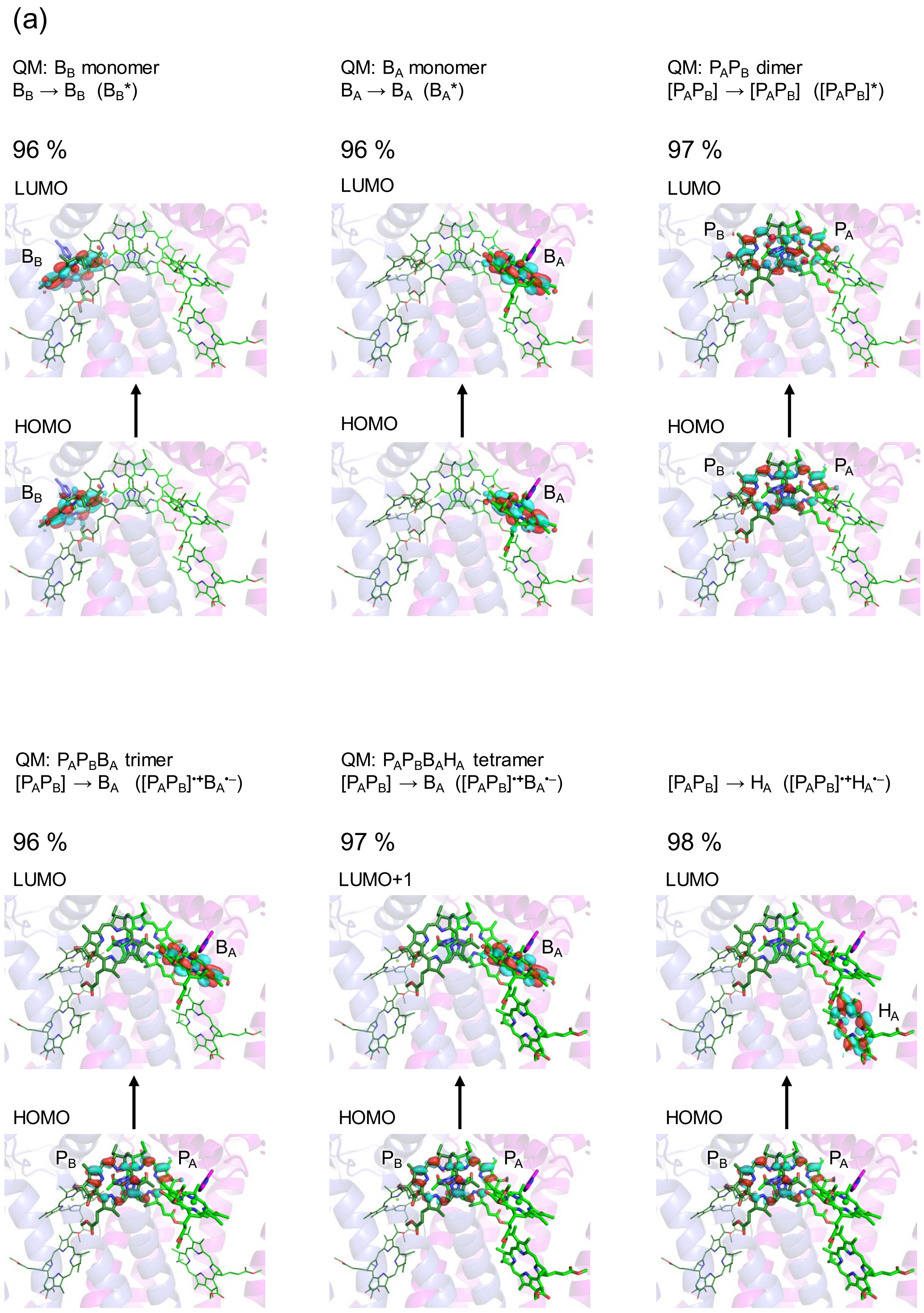

(a)
QM: B_B monomer
$B_B \rightarrow B_B$ (B_B*)
96 %
LUMO
B_B
HOMO
B_B
QM: B_A monomer
$B_A \rightarrow B_A$ (B_A*)
96 %
LUMO
B_A
HOMO
B_A
QM: P_AP_B dimer
$[P_AP_B] \rightarrow [P_AP_B]$ ($[P_AP_B]$*)
97 %
LUMO
P_B
P_A
HOMO
P_B
P_A
QM: $P_AP_BB_A$ trimer
$[P_AP_B] \rightarrow B_A$ ($[P_AP_B]^{\bullet +}B_A^{\bullet -}$)
96 %
LUMO
B_A
HOMO
P_B
P_A
QM: $P_AP_BB_AH_A$ tetramer
$[P_AP_B] \rightarrow B_A$ ($[P_AP_B]^{\bullet +}B_A^{\bullet -}$)
97 %
LUMO+1
B_A
HOMO
P_B
P_A
$[P_AP_B] \rightarrow H_A$ ($[P_AP_B]^{\bullet +}H_A^{\bullet -}$)
98 %
LUMO
H_A
HOMO
P_B
P_A

(b)

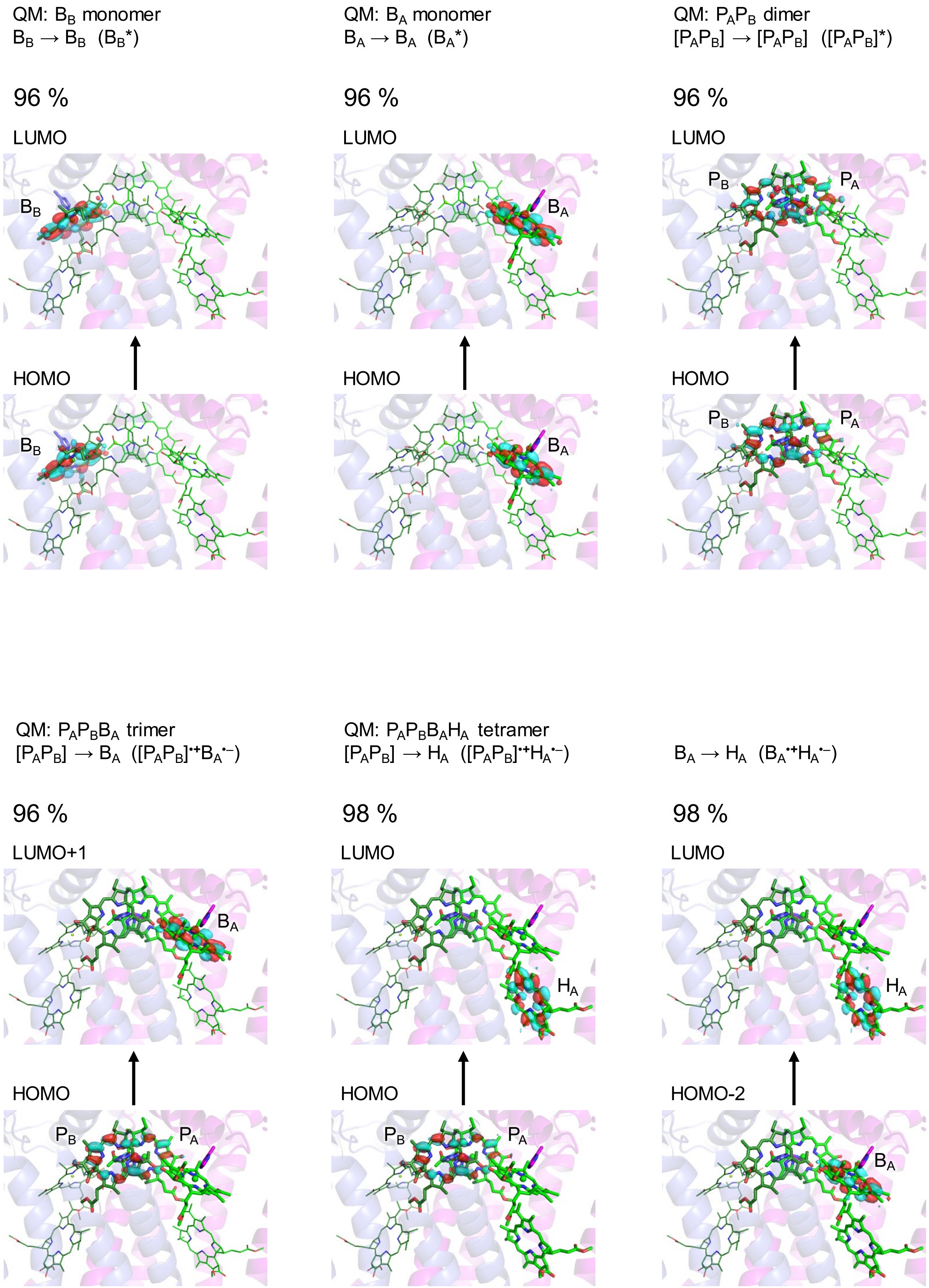
QM: B_B monomer
$B_B \rightarrow B_B$ (B_B*)
96 %
LUMO
B_B
HOMO
B_B
QM: B_A monomer
$B_A \rightarrow B_A$ (B_A*)
96 %
LUMO
B_A
HOMO
B_A
QM: P_AP_B dimer
$[P_AP_B] \rightarrow [P_AP_B]$ ($[P_AP_B]$*)
96 %
LUMO
P_B
P_A
HOMO
P_B
P_A
QM: $P_AP_BB_A$ trimer
$[P_AP_B] \rightarrow B_A$ ($[P_AP_B]^{\bullet+}B_A^{\bullet-}$)
96 %
LUMO+1
B_A
HOMO
P_B
P_A
QM: $P_AP_BB_AH_A$ tetramer
$[P_AP_B] \rightarrow H_A$ ($[P_AP_B]^{\bullet+}H_A^{\bullet-}$)
98 %
LUMO
H_A
HOMO
P_B
P_A
$B_A \rightarrow H_A$ ($B_A^{\bullet+}H_A^{\bullet-}$)
98 %
LUMO
H_A
HOMO-2
B_A

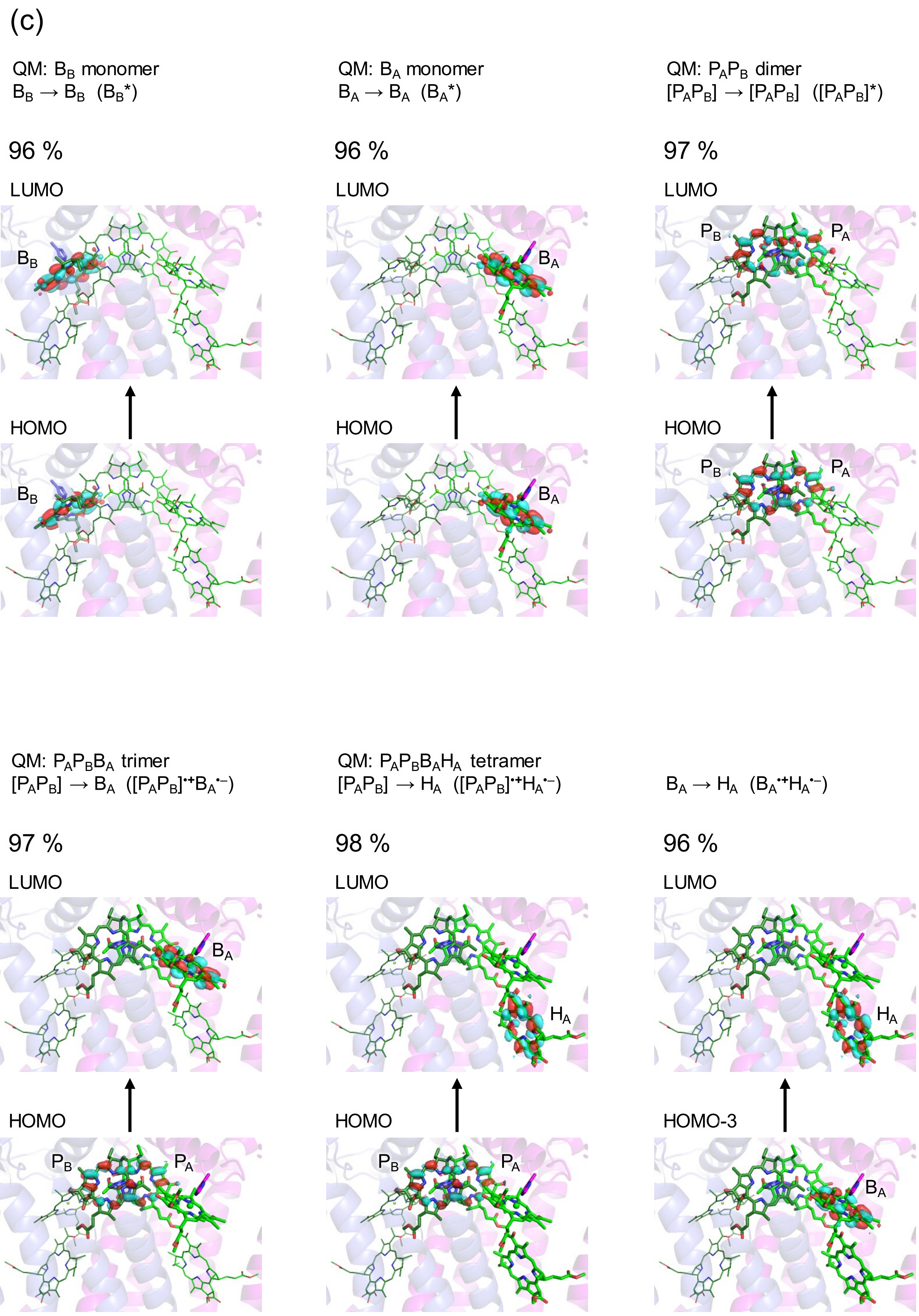
(c)
QM: B_B monomer
$B_B \rightarrow B_B$ (B_B*)
96 %
LUMO
B_B
HOMO
B_B
QM: B_A monomer
$B_A \rightarrow B_A$ (B_A*)
96 %
LUMO
B_A
HOMO
B_A
QM: P_AP_B dimer
$[P_AP_B] \rightarrow [P_AP_B]$ ($[P_AP_B]$*)
97 %
LUMO
P_B
P_A
HOMO
P_B
P_A
QM: $P_AP_BB_A$ trimer
$[P_AP_B] \rightarrow B_A$ ($[P_AP_B]^{\bullet +}B_A^{\bullet -}$)
97 %
LUMO
B_A
HOMO
P_B
P_A
QM: $P_AP_BB_AH_A$ tetramer
$[P_AP_B] \rightarrow H_A$ ($[P_AP_B]^{\bullet +}H_A^{\bullet -}$)
98 %
LUMO
H_A
HOMO
P_B
P_A
$B_A \rightarrow H_A$ ($B_A^{\bullet +}H_A^{\bullet -}$)
96 %
LUMO
H_A
HOMO-3
B_A

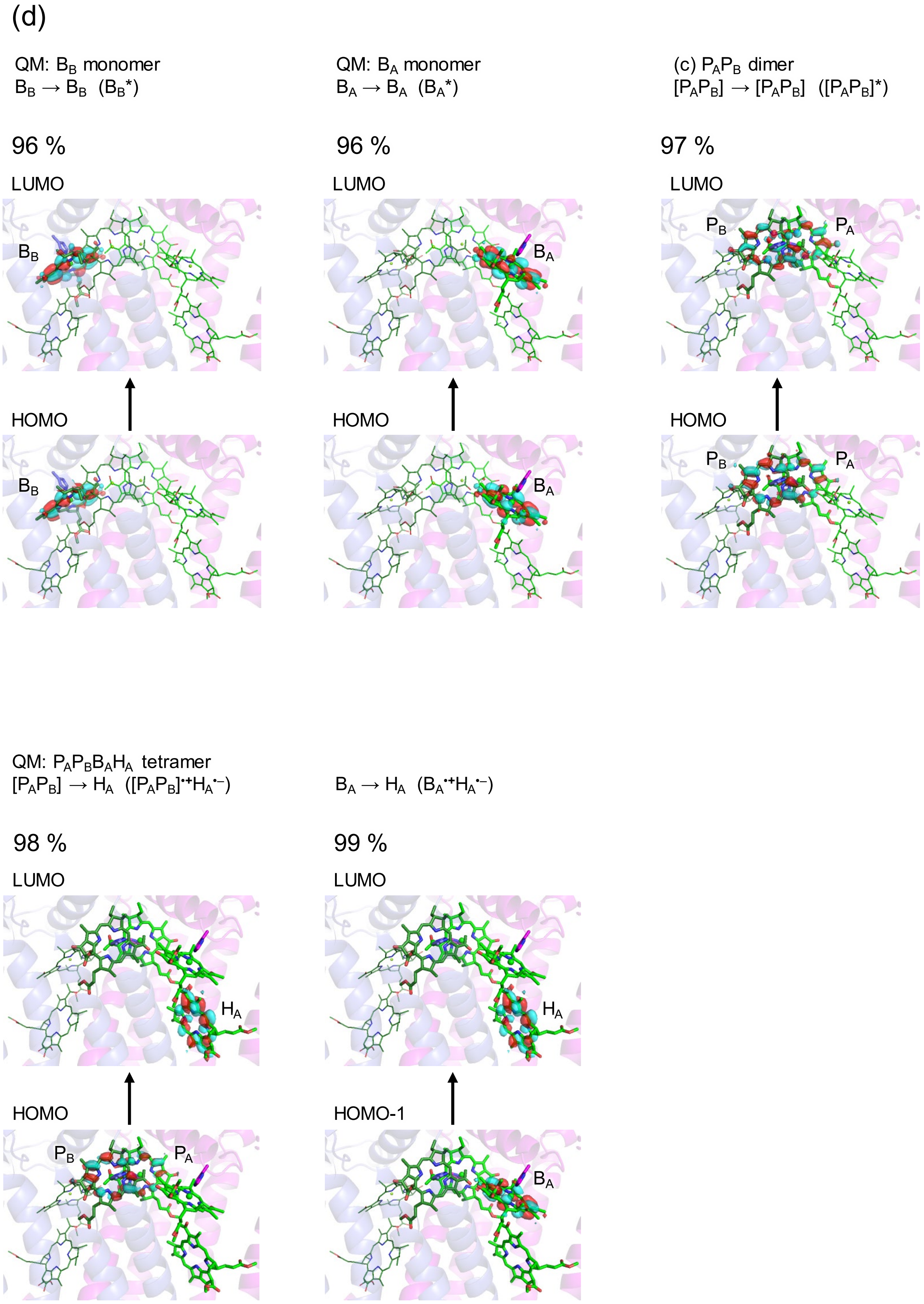

Figure S2. Orbital characteristics of charge-transfer excited states relevant to initial charge separation. (a) Tyr-OH...B_A and H_A-CH_3...Mg^{2+}-B_A conformation (Figure 2a). (b) Tyr-OH...P_B and H_A-

CH_3...Mg^{2+}-B_A conformation (Figure 2b). (c) Tyr-OH...B_A and H_A-C=O...Mg^{2+}-B_A conformation (Figure 2c). (d) Tyr-OH...P_B and H_A-C=O...Mg^{2+}-B_A conformation (Figure 2d).

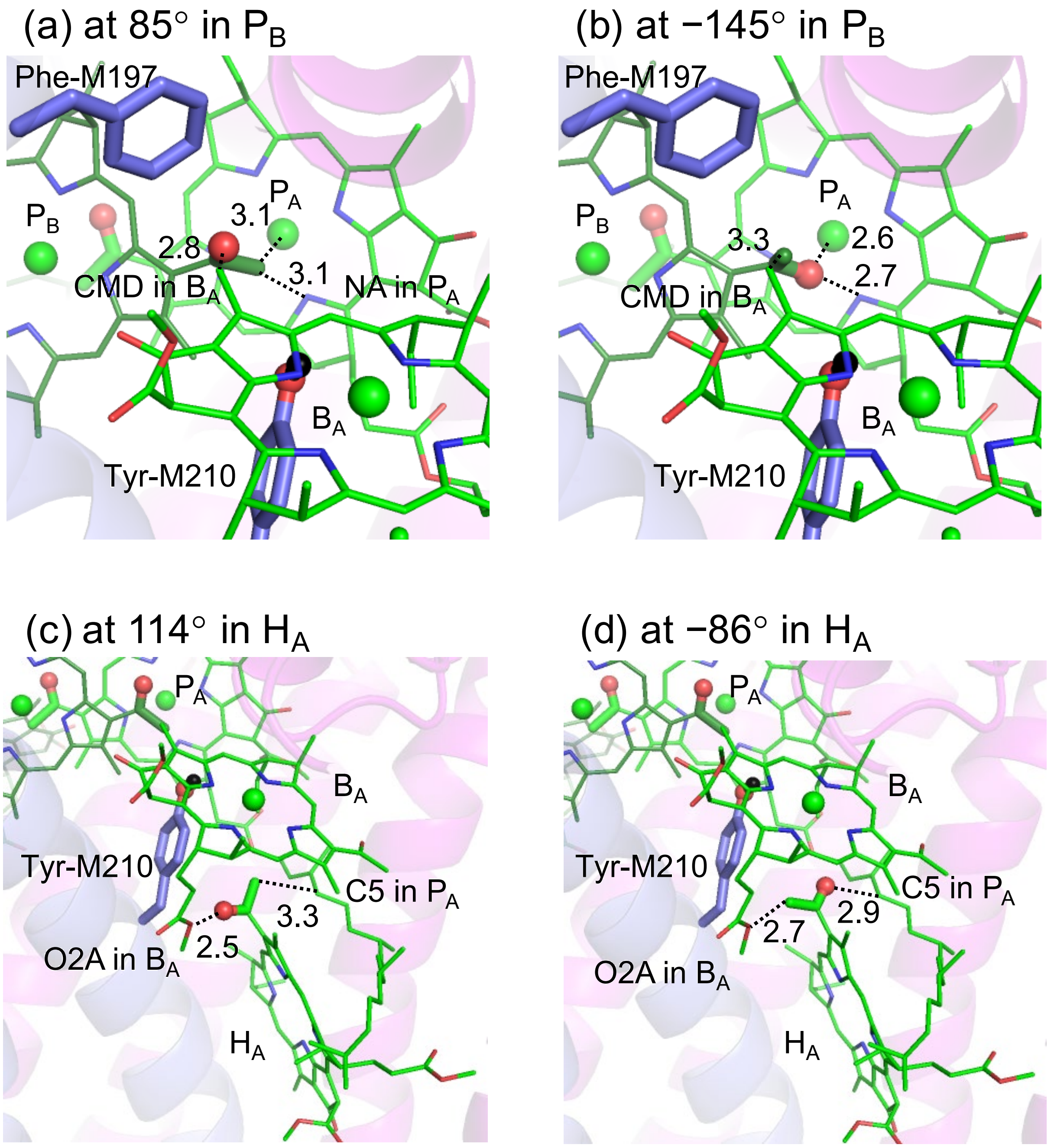

Figure S3. QM/MM-optimized intermediate conformations at rotational energy maxima obtained by constraining the methyl-keto dihedral angle (OBB…CAB…C3B…C4B). (a) Conformation at the energy maximum at 85° in the P_B methyl-keto orientation (right peak in Figure 4b). (b) Conformation at the energy maximum at −145° in the P_B methyl-keto orientation (left peak in Figure 4b). (c) Conformation at the energy maximum at 114° in the H_A methyl-keto orientation (right peak in Figure 5a). (d) Conformation at the energy maximum at −86° in the H_A methyl-keto orientation (left peak in Figure 5a).

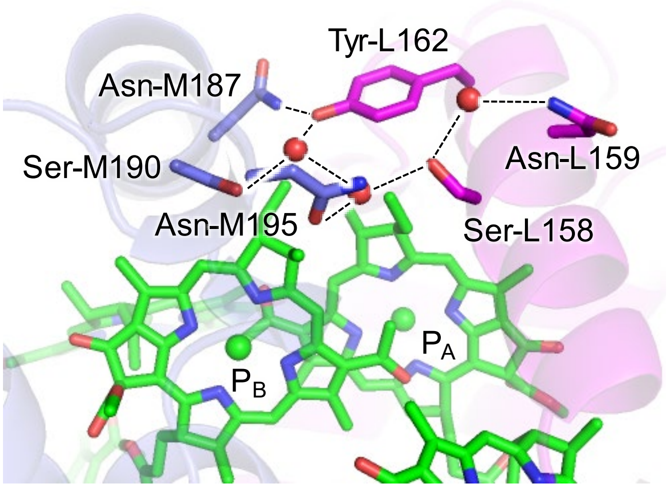

Figure S4. Orientations of side chains of Asn-M187 and Asn-L159 near P_AP_B in the crystal 1.87 Å structure (PDB code: 2J8C). Dotted lines indicate H-bonds.
